# Kallikrein-related peptidase 7 is a potential target for the treatment of pancreatic cancer

**DOI:** 10.18632/oncotarget.24132

**Published:** 2018-01-10

**Authors:** Jian Ping Du, Lin Li, Jun Zheng, Ding Zhang, Wei Liu, Wei Hong Zheng, Xiao Song Li, Ru Cheng Yao, Fangyu Wang, Sen Liu, Xiao Tan

**Affiliations:** ^1^ Institute of Hepatopancreatobilary Surgery, China Three Gorges University, Yichang 443003, P.R. China; ^2^ The First College of Clinical Medical Science, China Three Gorges University, Yichang, 443003, P.R. China; ^3^ Hubei Key Laboratory of Tumor Microenvironment and Immunotherapy, Medical School of China Three Gorges University, Yichang 443002, P.R. China; ^4^ National “111” Center for Cellular Regulation and Molecular Pharmaceutics, Hubei University of Technology, Wuhan 430068, P.R. China; ^5^ Department of Vascular surgery, Xianning Central Hospital, The First Affiliated Hospital of Hubei University of Science and Technology, Xianning, 437100, P.R. China; ^6^ College of Life Science and Environment, Hengyang Normal University, Hengyang, 421008, P.R. China

**Keywords:** pancreatic ductal adenocarcinoma, kallikrein-related peptidase 7, short hairpin RNA, small organic inhibitor, chemotherapy target

## Abstract

Pancreatic cancer is one of the deadliest cancers with very poor prognosis, and the five-year survival rate of the patients is less than 5% after diagnosis. Kallikrein-related peptidases (KLKs) belong to a serine protease family with 15 members that play important roles in cellular physiological behavior and diseases. The high expression level of KLK7 in pancreatic cancer tissues is considered to be a marker for the poor prognosis of this disease. In this work, we set out to investigate whether KLK7 could be a target for the treatment of pancreatic cancer. Short hairpin RNAs (shRNAs) were designed and constructed in lentivirus to knock down KLK7 in pancreatic cancer cell line PANC-1, and the real time cellular analysis (RTCA) was used to evaluate cell proliferation, migration and invasion abilities. Small molecules inhibiting KLK7 were discovered by computer-aided drug screening and used to inhibit PANC-1 cells. Our results confirmed that KLK7 is significantly up-regulated in pancreatic cancer tissue, and knocking down or inhibiting KLK7 efficiently inhibited the proliferation, migration and invasion of pancreatic cancer cells. This study suggested that KLK7 could be a potential chemotherapy target for treatment of pancreatic cancer, which would provide us a novel strategy for the treatment of this disease.

## INTRODUCTION

Pancreatic ductal adenocarcinoma (PDAC) is one of the deadliest solid tumors with a 5-year survival rate less than 5% and the median survival period after diagnosis is about 6 months [1, 2]. PDAC has a very high tendency for local invasion and distant metastases. After diagnosis, only about 20% of PDAC patients have the possibility of surgical resection. Even for these patients, the 5-year survival rate is still at a very low level due to the early recurrence or metastatic spread of the tumors [3]. For PDAC patients with local invasion and distant metastases, the therapy trials, including chemotherapy and radiotherapy, could not significantly improve the overall survival time. Therefore, developing more specific and efficient new drugs for PDAC has important clinical value.

Human tissue kallikrein-related peptidases (KLKs) are a group of serine proteases and mainly synthesized and secreted by epithelial cells [4]. KLKs are involved in extracellular matrix remodeling, skin desquamation, hormone precursor processing and regulation of tumor cell proliferation by activating growth factor and hydrolyzing growth factor binding proteins [5]. It has been reported that several KLK members were dysregulated in cancers and correlated with tumor development [6, 7].

KLK7 was originally found in skin and it has important roles in stratum corneum formation and skin desquamation [8]. KLK7 is over expressed in pancreatic cancer tissues, and the expression level of KLK7 could be an important prognostic indicator in different cancers including colon cancer, ovarian cancer and pancreatic cancer [9–11]. KLK7 secreted by tumor cells or added exogenously could promote the degradation of extracellular matrix (ECM) and cellular adhesive molecules (CAMs) such as fibronectin, desmoglein 2, E-cadherin, urokinase-type plasminogen activator receptor. As a result, KLK7 reduces the aggregation of pancreatic cancer cells and enhance their migration [12–15]. However, whether KLK7 has the potential to be a therapeutic target for the treatment of pancreatic cancer has not been studied very well. In this work, we investigated the potential of KLK7 as a therapeutic target of pancreatic cancer. The results showed that over-expressed KLK7 promoted the proliferation, migration and invasion of pancreatic cancer cells. Down-regulating KLK7 expression or inhibiting KLK7 activity by small molecule inhibitors developed in this study could inhibit the growth, migration and invasion of pancreatic cells. Our results suggest that KLK7 could be a potential chemotherapy target for pancreatic cancer.

## RESULTS

### KLK7 is up-regulated in pancreatic cancer

**Figure 1:**
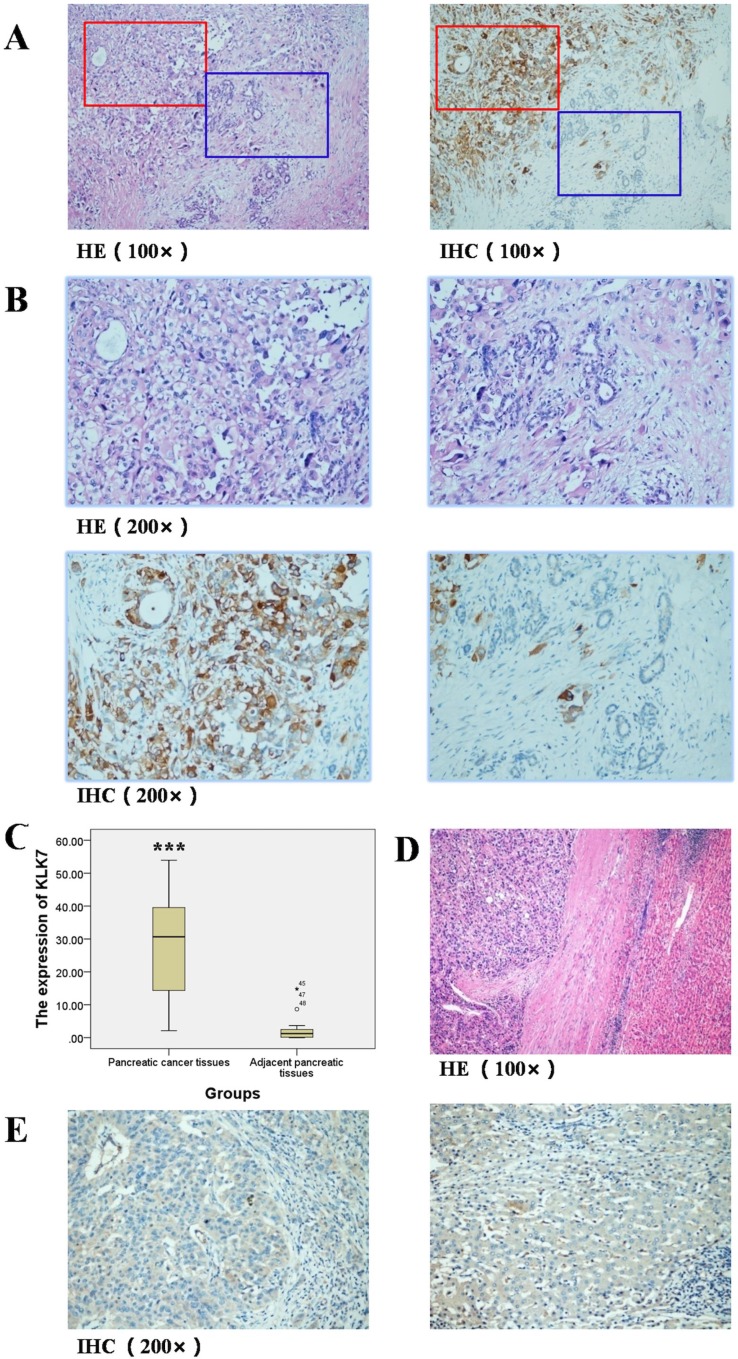
Expression of KLK7 in pancreatic cancer tissues and adjacent pancreatic tissues, hepatocellular carcinoma tissues and adjacent liver tissues (**A**) HE and IHC staining of KLK7 protein in pancreatic cancer and adjacent pancreatic tissues (Representative image, 100×); (**B**) HE and IHC staining of KLK7 protein in pancreatic cancer and adjacent pancreatic tissues (Representative image, 200×), expression of KLK7 in pancreatic cancer tissues was up-regulated (reddish brown), but almost absent in the adjacent normal pancreatic tissues; (**C**) semi-quantitative study of expression of KLK7 in pancreatic cancer and adjacent pancreatic tissues, the relative expression of KLK7 protein in pancreatic cancer is 30-fold higher than that in adjacent pancreatic tissues; (**D**) HE staining of hepatocellular carcinoma tissues and the corresponding adjacent normal liver tissues (Representative image, 100×); (**E**) IHC staining of KLK7 protein in hepatocellular carcinoma and adjacent liver tissues (Representative image, 200×), the expression of KLK7 is unchanged in both of the hepatocellular carcinoma tissues and the corresponding adjacent normal liver tissues. ^***^P < 0.001.

The paraffin-embedded specimens from 49 PDAC patients were analyzed by HE and IHC (Figure 1A, 1B). Low expression of KLK7 was observed in the tumor adjacent tissues, whereas in the cancerous tissues, elevated expression of KLK7 up to 30 folds as evaluated by the mean IOD values (*P* < 0.001 = was noticed (Figure 1C). However, the expression level of KLK7 had no such elevation in hepatocellular carcinoma tissues as control (Figure 1D, 1E).

### Cell proliferation, migration and invasion are decreased in KLK7-silenced PANC-1 cells

**Figure 2:**
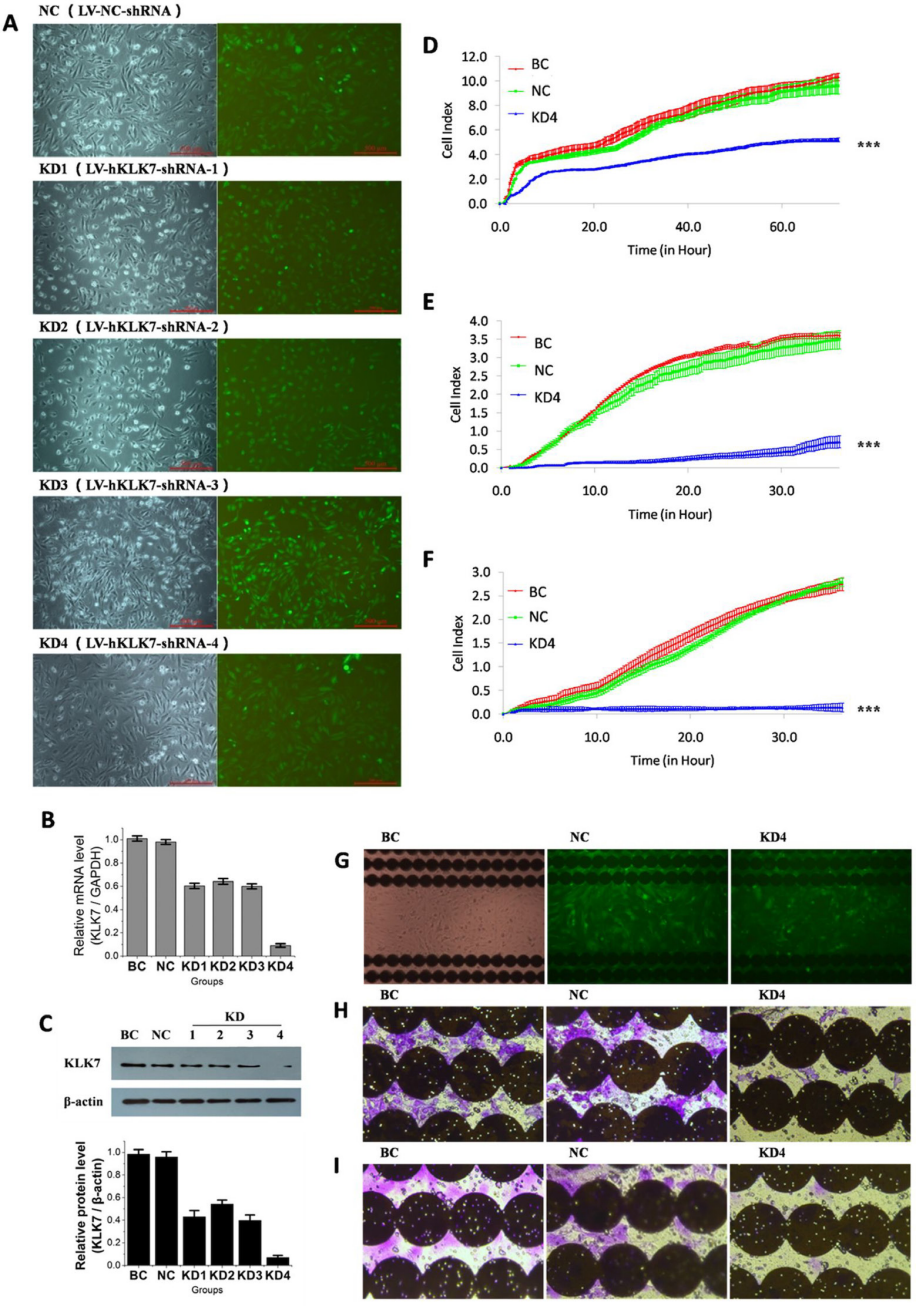
Infection of pancreatic cancer PANC-1 cells by lentivirus to knock down KLK7 expression, and compact of KLK7 gene silencing on PANC-1 cell proliferation, migration and invasion abilities identified by RTCA and microscopy (**A**) observation of PANC-1 cells infected by lentivirus under microscopy. KD1, KD2, KD3, KD4 and NC are PANC-1 cells infected with LV-hKLK7-shRNA-1, -2, -3, -4 and LV-NC-shRNA respectively, BC are uninfected PANC-1 cells; (**B**) expression of KLK7 mRNA of PANC-1 cells in each group evaluated by real-time PCR, the relative expression of KLK7 mRNA in KD1-4 groups were decreased; (**C**) expression of KLK7 protein of PANC-1 cells in each group evaluated by Westernblot, the relative expression of KLK7 protein in KD1-4 groups were significantly decreased; (**D)** and (**G**) proliferation assay of PANC-1 cells in BC, NC and KD4 group. The proliferation ability of KD4 group was decreased compared to BC and NC groups; (**E)** and (**H**) migration assay of PANC-1 cells in BC, NC and KD4 group, migration ability of KD4 group was decreased compared to BC and NC groups; (**F**) and (**I**), matrigel invasion assay of PANC-1 cells in BC, NC and KD4 group, invasion ability of KD4 group was decreased compared to BC and NC groups,; the photographs were taken at the end of the RTCA assays. ^***^P < 0.001.

To study the role of KLK7 in pancreatic cancer, we established KLK7 silenced PANC-1 cells by four specific shRNAs (verified by BLAST) using lentivirus (Figure 2A). PANC-1 cells infected by recombinant lentivirus expressed GFP proteins after 24 h of the infection, and the intensity of the fluorescence increased gradually over time and reached the maximal peak at 72 h. After selected by puromycin for 5 days, the stably transfected cell lines were obtained. The PANC-1 cell line infected with LV-NC-shRNA was named as negative control (NC), the cell lines infected with LV-hKLK7-shRNA-1, -2, -3 or -4 were named as KLK7 knocked down 1, 2 3 or 4 (KD1, 2, 3 or 4), and the untreated PANC-1 cell line was named as the blank control (BC). The morphological observation showed that, unlike BC or NC cells, the KLK7 silenced PANC-1 cells are more likely to form clusters, especially for KD4 cells (Supplementary Figure 1). Analysis of the mRNA and protein levels by Real-time PCR and western blotting assay confirmed that the expression of KLK7 in KD1-4 groups were down regulated at both transcriptional and translational levels (Figure 2B, 2C). For KD4 group, the expression of KLK7 is decreased by around 90% compared to NC group. According to these results, KD4 was then chosen for the followed analyses.

In the proliferation assay (Figure 2D, 2G), no significant difference was noticed between the BC and NC groups. However, the proliferation rate of KD4 cells was very low, and it decreased about 50% compared to BC and NC groups (*P* < 0.001=. In the migration assay (Figure 2E, 2H), no significant difference was recorded between the BC and NC groups. But the migration rate of KD4 group was obviously low, which decreased about 85% compared to BC and NC groups (*P* < 0.001=. Similar observation has been obtained in the invasion assay (Figure 2F, 2I). The invasion capacity of KD4 was almost fully inhibited (*P* < 0.001=. As negative control, KLK7 silenced hepatocellular carcinoma HepG2 cells were established, but our data showed that unlike PANC-1 cells, the knocking down of KLK7 did not affect the proliferation, migration, and invasion of HepG2 cells (Supplementary Figure 2), demonstrating that our designed LV-hKLK7-shRNA-4 do not target other proteins.

### The proliferation, migration, and invasion capacity of the KD4 cells were recovered by exogenous KLK7

**Figure 3:**
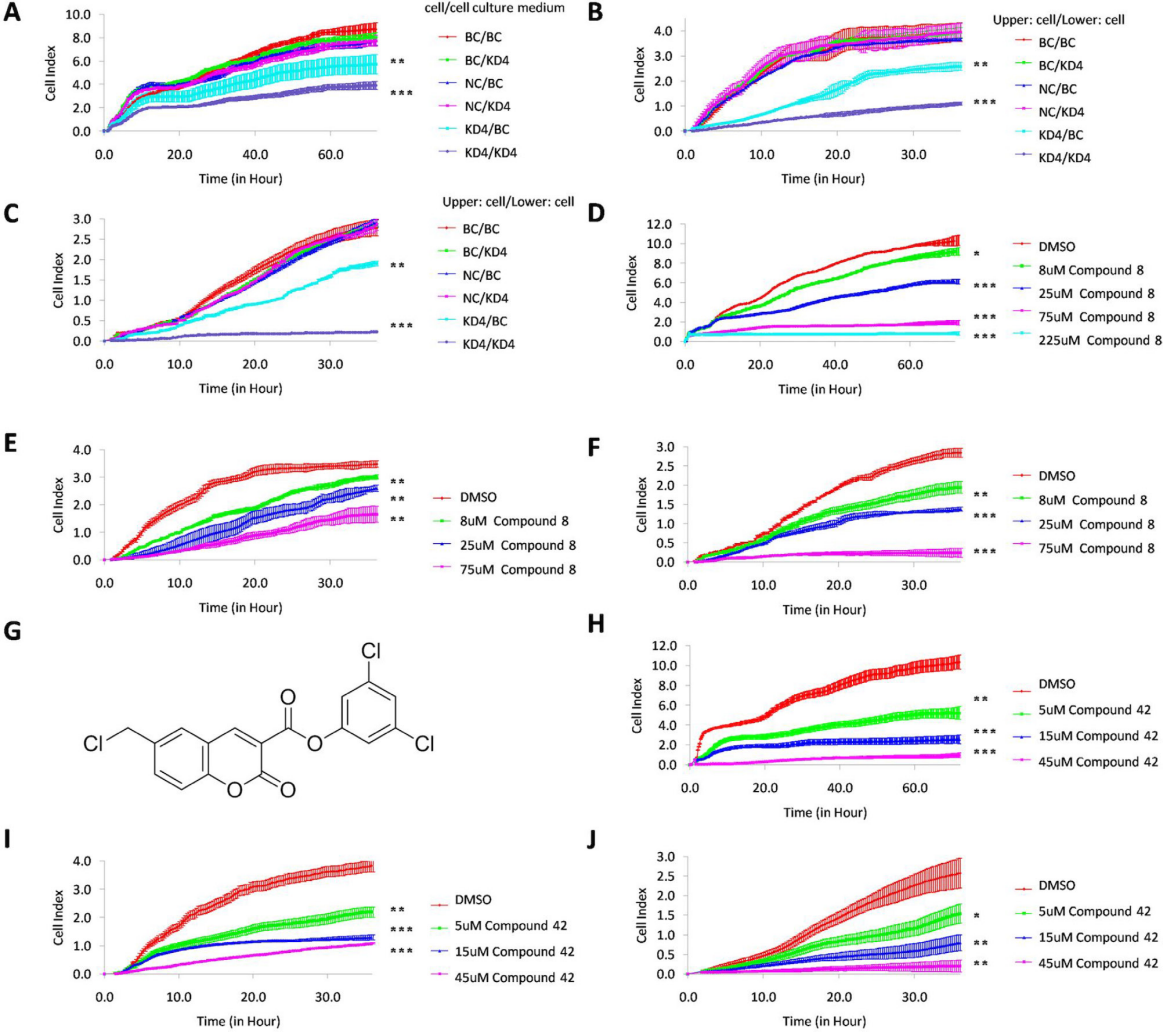
Recovery of KD4 cell proliferation, migration and invasion abilities by exogenous KLK7, and inhibition of PANC-1 cell proliferation, migration and invasion ability by KLK7 inhibitor (compound 8) issued from virtual screening or KLK7 suicide inhibitor (compound 42) issued from previous study (**A**) proliferation curve of PANC-1 cells in BC/BC culture supernatant, BC/KD4 culture supernatant, NC/BC culture supernatant, NC/KD4 culture supernatant, KD4/BC culture supernatant and KD4/KD4 culture supernatant group, where PANC-1 cells in BC, NC or KD4 groups were treated by PANC-1 cell culture medium with (BC culture supernatant) or without (KD4 culture supernatant) exogenous KLK7. Proliferation ability of KLK7 silenced PANC-1 cells in KD4/BC culture supernatant group was increased compared to that of KD4/KD4 culture supernatant group. (**B**) migration curve of PANC-1 cells in BC/BC, BC/KD4, NC/BC, NC/KD4, KD4/BC and KD4/KD4 group, where PANC-1 cells of BC, NC or KD4 in the upper chamber of plate were treated by exogenous KLK7 secreted by PANC-1 cells in BC group or KD4 group from the lower chamber of plate. The migration ability of KLK7 gene silenced PANC-1 cells in KD4/BC group was increased compared to that of KD4/KD4 group. (**C**) matrigel invasion curve of PANC-1 cells in BC/BC, BC/KD4, NC/BC, NC/KD4, KD4/BC and KD4/KD4 group, where PANC-1 cells of BC, NC or KD4 in the upper chamber of plate were treated by exogenous KLK7 secreted by PANC-1 cells in BC group or KD4 group from the lower chamber of plate. The invasion ability of KLK7 silenced PANC-1 cells in KD4/BC group was increased compared to that of KD4/KD4 group. Proliferation (**D**), migration (**E**) and invasion (**F**) abilities of PANC-1 cells were significantly inhibited by increasing compound 8 concentration (8 μM, 25 μM, 75 μM, 225 μM for proliferation assay and 8 μM, 25 μM, 75 μM for migration and invasion assay). (**G**) molecular structure of compound 42. Proliferation (**H**), migration (**I**) and invasion (**J**) abilities of PANC-1 cell were significantly inhibited by increasing compound 42 concentration (5 μM, 15 μM and 45 μM). DMSO was used as negative control. ^*^P < 0.05, ^**^P < 0.01, ^***^P < 0.001.

As KLK7 is a protease which can be secreted into intercellular space, we tested whether the proliferation, migration, and invasion capacities inhibited by KLK7 silencing could be recovered using exogenous KLK7. In the proliferation assay (Figure 3A), the culture supernatants from BC, NC or KD4 cells were used as the source of exogenous KLK7 and the results showed that there was no significant difference between the BC/BC culture supernatant, NC/BC culture supernatant, BC/KD4 culture supernatant and NC/KD4 culture supernatant groups, suggesting that the culture supernatant had no impact on the proliferation capacity of the control PANC-1 cells. However, the proliferation rate of KD4 cells in KD4/BC culture supernatant group was significantly increased compared with that in KD4/KD4 culture supernatant group (*P* < 0.05). In the migration assay and invasion assay (Figure 3B, 3C), similar observations had been obtained. The migration and invasion rate of the KD4 cells in KD4/BC group was increased compared with that in the KD4/KD4 group (*P* < 0.001). This result demonstrated that KLK7 secreted by PANC-1 control cells in the lower chamber could increase the migration capacity and especially invasion capacity of KD4 cells in the upper chamber. Taking together, these results suggested that KLK7 could be a target to inhibit pancreatic cancer development.

### *In silico* screening preparation

**Figure 4:**
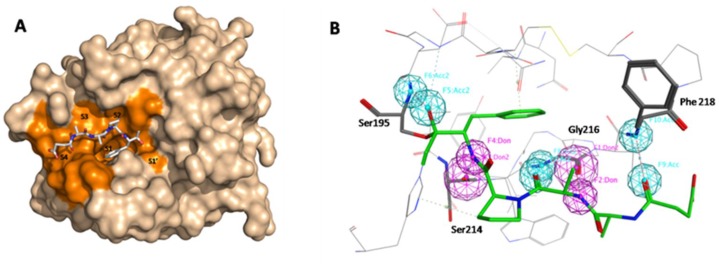
*In silico* screening preparation (**A**) Druggable pocket identified by DoGSiteScorer using the crystal structure of KLK7 (2QXH). The druggable zone in the region of the active site involves the active site specificity sub-pockets S’1 and S1-S4 (in orange). The ligand Suc-Ala-Ala-Pro-Phe-chloromethylketone co-crystallized within the active site is shown in a stick representation. (**B**) pharmacophore model builded in MOE. Four hydrogen bonds formed between KLK7 and its ligand: P1-Phe N and Ser214 O, P3-Ala N and Gly216 O, P3-Ala O and Gly216 N, succinyl O and Phe218 N, P1-Phe O and Ser195 N.

To investigate whether KLK7 is an efficient target for the chemotherapy of pancreatic cancer, specific and efficient KLK7 inhibitors are needed. In this experiment, we used computer aided drug screening to screen non-covalent inhibitors of KLK7. Firstly, we analyzed the crystal structure of KLK7 (2QXH) using DogSiteScorer server to identify the druggable region at the surface of the protein. The result showed that the most potent druggable regions were localized at the active site of KLK7 including the sub-site S’1, S1-S4 (Figure 4A), which had a druggability score of 0.79 (0–1). This site was then chosen for pharmacophore model building and docking. In the pharmacophore generation, hydrogen bond was carefully considered, since four hydrogen bonds were formed between KLK7 and its peptide ligand Suc-Ala-Ala-Pro-Phe-chloromethylketone: P1-Phe N and Ser214 O, P3-Ala O and Gly216 N, P3-Ala N and Gly216 O, succinyl O and Phe218 N [16]. In addition, we also found that one hydrogen bond could form between P1-Phe O and Ser195 N. Thus, to build the pharmacophore model in MOE, following characteristic elements are important: three hydrogen bond acceptor centers at P1-Phe O, P3-Ala O and succinyl O; two hydrogen bond donor centers: P3-Ala N and P1-Phe N (Figure 4B).

### *In silico* screening of novel KLK7 inhibitors

**Figure 5:**
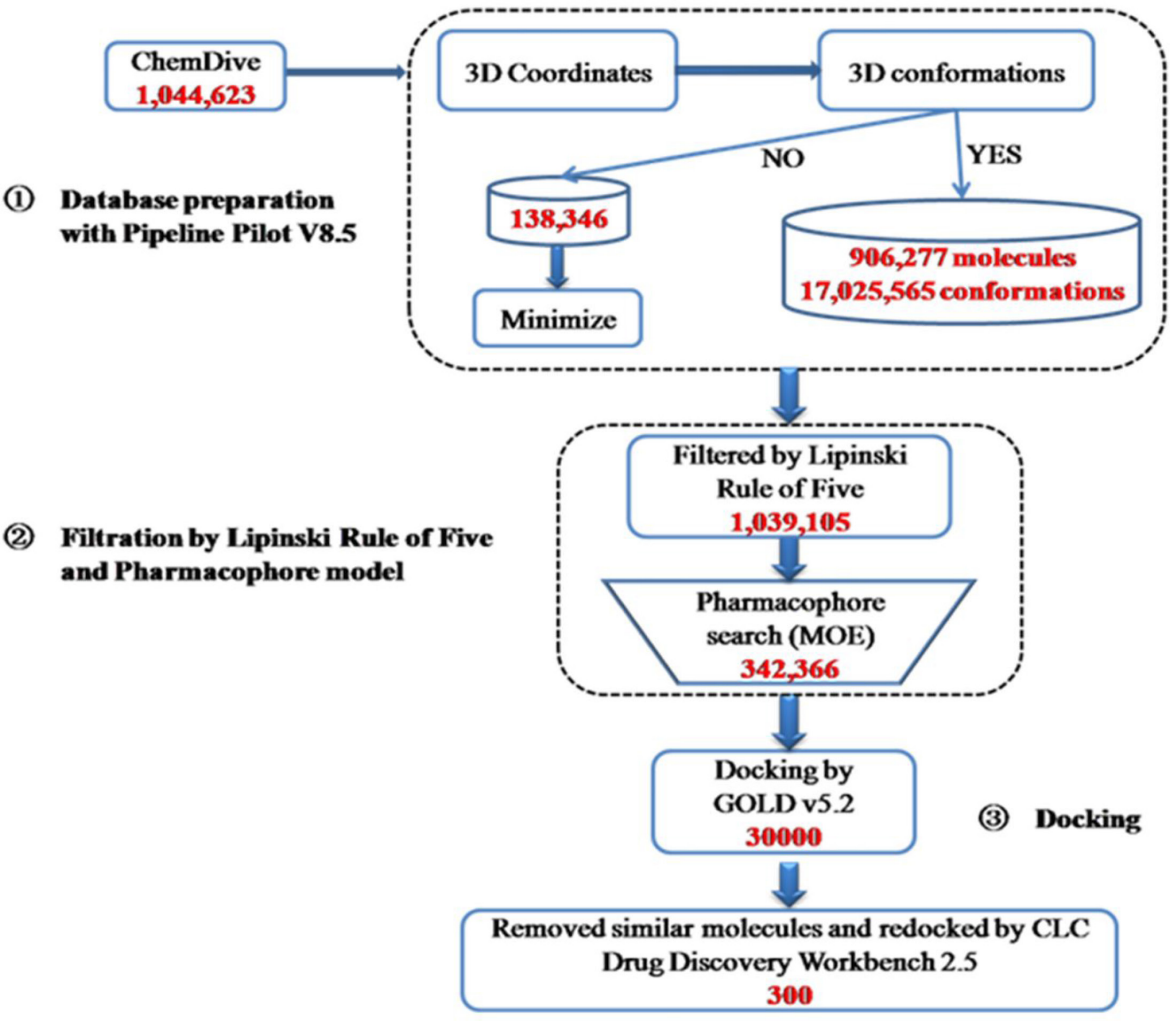
Summary of the virtual screening protocols used to search inhibitors of KLK7 1,044,623 compounds in Chemdiv library were used to generate 3D-conformations, and then filtered by Lipinski Rule of Five and pharmacophore model of KLK7. The structure-based virtual screening using GOLD and CLC Drug Discovery Workbench software was performed and a total of 300 hits were obtained.

The 1,044,623 molecules in the ChemDiv database were prepared in Pipeline Pilot to generate 3-D conformations and minimized (Figure 5). Then, the obtained 17,163,911 conformations were filtered by Lipinski Rule of Five to remove the molecules without druggable properties. 95% of the conformations were removed as non druggable molecules and 1,039,105 conformations were kept for phamacophore search. After phamacophore filtration, 696,739 conformations were removed, as they do not form enough hydrogen bonds with KLK7 to ensure their interaction. The rest 342,366 conformations were kept for docking screening. In the docking step, GOLD was first used for flexible molecular docking, and the 342,366 conformations were ranked by their docking scores. The top 10% of the conformations with the highest score (30,000 conformations) were selected. After removing the conformations with the same molecular structure, the CLC Drug Discovery Workbench was used to re-evaluate these conformations. Finally, a total of 300 molecules (the top 300 of the conformations with the highest PLANTS_PLP_ score) were kept for subsequent experimental screening.

### Experimental validation of novel KLK7 inhibitors

**Figure 6:**
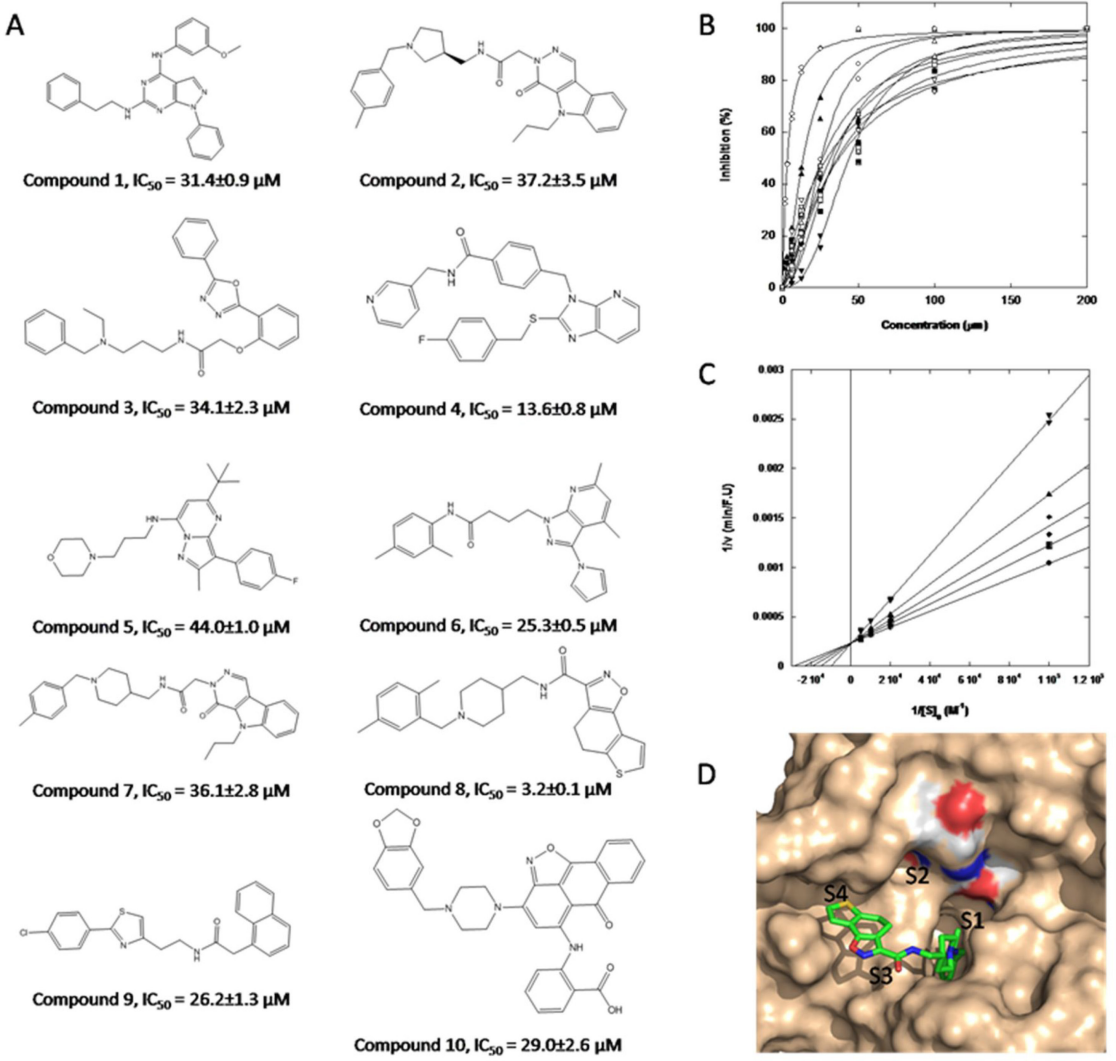
Structure of hits obtained through virtual screening and their inhibitory properties towards KLK7 (**A**) molecular structure and inhibitory potency of 10 compounds that inhibit KLK7; (**B**) measurement of IC_50_ of the 10 hits toward KLK7, which vary from 3.2 μM to 44 μM. (●) Compound 1; (■) Compound 2; (♦)Compound 3; (▲) Compound 4; (▼) Compound 5; (○)Compound 6; (□)Compound 7; (△)Compound 8; (▽)Compound 9; (◇)Compound 10; the most potent inhibitory molecule was compound 8 (IC_50_ = 3.2 μM); (C) Lineweaver-Burk plot for KLK7 at different concentrations of compound 8: (●) 0; (■) 0.5 μM; (◆) 1 μM; (▲) 2 μM; (▼) 4 μM; (D) Compound 8 docked into the KLK7 catalytic site with its predicted binding modes. The enzyme surface is colored light brown and the ligand is shown in stick representation. The catalytic triad (His57, Asp102, Ser195) is colored by element. Figure was prepared with Pymol.

The 300 compounds purchased from the ChemDiv library were evaluated for the *in vitro* inhibition of KLK7 by measuring the hydrolysis of the FRET substrate MCA-Arg-Pro-Lys-Pro-Val-Glu-Nval-Trp-Arg-Lys(Dnp)-NH_2_. Ten compounds fully inhibited KLK7 activity at 200 μM (Figure 6A). The IC_50_ values of these 10 inhibitors varied from 3 μM to 44 μM (Figure 6B), among which the compound 8 had the best inhibitory potency against KLK7 with IC_50_ = 3 μM. The dilution assay showed that all compounds should be non-covalent inhibitors since their inhibition was reversible. Lineweavere-Burk double reciprocal plots showed that the compound 8 was a competitive inhibitor with Ki = 3.6 μM (Figure 6C).

The putative binding mode of compound 8 on KLK7 was shown in Figure 6D modeled from GOLD and CLC drug Discovery Workbench. The scaffold of the compound 8 is rather flexible with 6 rotatable bonds, although two of them are constrained by delocalized π bonds. KLK7 belongs to the chymotrypsin serine protease, and the S1 site of KLK7 is hydrophobic. As expected, the di-substituted phenyl group of compound 8 protrudes deeply into the S1 site of KLK7, and the rest part goes towards to the S3 and S4 sites.

### Compound 8 inhibited PANC-1 cell proliferation, migration and invasion

The inhibitory potency of compound 8 against PANC-1 cells was evaluated by RTCA. In the proliferation assay (Figure 3D), the cell index values of PANC-1 cells treated by different concentrations of compound 8 were significantly lower than that of the negative control (*P* < 0.05=. Moreover, with the increase of the concentration of compound 8 (8 μM, 25 μM, 75 μM and 225 μM), the inhibitory potency against PANC-1 increased (*P* < 0.05=. When the concentration of compound 8 was increased to 225 μM, the proliferation ability of PANC-1 was almost 100% inhibited (*P* < 0.001=. In the migration and matrigel invasion assays, similar results were observed (Figure 3E, 3F). The migration and invasion ability of PANC-1 cells was inhibited by compound 8 in a concentration depend manner (*P* < 0.05). The results were in good agreement with the data from the KD4 cells. The same findings were observed by using a covalent suicide inhibitors of KLK7 (compound 42, Figure 3G) identified previously (Figure 3H–3J).

## DISCUSSION

PDAC is one of the most lethal solid tumor [1] with approximately 80% of pancreatic cancer patients cannot undergo a curative operation [3]. The current therapy of PDAC is not satisfactory, and novel therapeutic targets and drugs would be very valuable [17, 18]. Migration and invasion are the most critical aspect of tumor development, and serine proteases play vital roles in these processes [19].

KLKs are a group of serine proteases aberrantly expressed in numerous tissues including cancer [20]. KLKs are key players in promoting cancer progression, and have great potential in KLK-based therapy [21]. KLK7 is over-expressed in PDAC tissues and could be used as an important marker in the prognosis of PDAC [11]. The loss of cell-cell and cell-ECM adhesion allows tumor cells to detach from the primary tumor mass and penetrate through the surrounding tissue and basement membrane to disseminate. Over expression of KLK7 or exogenous addition of KLK7 contributes to tumor cell migration and facilitates tumor cell invasion through releasing individual cells by cleaving junction proteins or ECM to allow movement of the pancreatic cancer cells [12, 13].

In our study, we showed for the first time that the expression of KLK7 is elevated up to 30 folds in PDAC tissue of Chinese patients. Meanwhile, we discovered that knocking down of KLK7 in PANC-1 cells resulted in decrease in cell proliferation, migration and invasion abilities. The molecular mechanisms associated with KLK7 silencing-induced anti-migration and anti-invasion abilities identified in our study can be interpreted as the cleavage of ECM proteins and junction/adhesion molecules by KLK7 [14, 15]. We should also point out that compared to migration assay, in the matrigel invasion assay, no KD4 cells could pass the matrigel moving to the lower chamber of the plate, indicating that silencing KLK7 could more efficiently inhibit the invasion ability rather than the migration ability of PANC-1, and KLK7 might help the degradation of ECM

In our study, anti-proliferative effect of silencing KLK7 on PANC-1 cells has also been observed, but the underlying mechanism has not been elucidated yet. It’s possible that the anti-proliferation induced by silencing KLK7 acts through protease-activated receptors-dependent proliferative pathways [22, 23], or the regulation of androgen-mediated proliferation pathways [24] and growth factor-mediated pathways [9], since these proliferation pathways are involved in several cancers where KLKs are deregulated. It is highly probable that these mechanisms are involved in our case, but further studies are needed to confirm this hypothesis.

Modification of the tumor microenvironment is essential for cancer progression, and according to the seed and soil theory, a pro-tumoral microenvironment is necessary for tumor establishment and progression [25]. During pancreatic cancer progression, KLK7 is produced by cancer cells and released into the microenvironment [26]. In our study, the cell culture medium from wild type PANC-1 could significantly increase the migration and invasion capacities of KD4 cell, indicating that KLK7 can act as a paracrine factor on other cells located at the tumor microenvironment and regulate the malignant progress of cancer cells.

KLK7 was originally found in skin and presented as a potential target for treatment of skin disease, and its activity can be readily blocked by small molecule inhibitors [27]. Our study suggests that KLK7 can be considerate as a novel target for anti-pancreatic cancer drug design. Since KLK7 is mainly expressed in skin tissues, targeting KLK7 in PDAC tissues is relative specific and would be safe. Moreover, silencing KLK7 activity leads to PANC-1 cell regression and decreases the malignant progression of PDAC. However, it was reported that Kallikrein-related peptidase 7 was down-regulated in prostate cancer [28], indicating that inhibition of KLK7 may influence the normal function of prostate, but this needs further investigations.

Computer-aided drug screening tools have become effective and indispensable tools in chemotherapy development [29]. They will help us to reduce the long process in drug discovery and the cost. In this study, we discovered ten non-covalent inhibitors of KLK7 with different molecular structures. Among these ligands, one compound (compound 8) inhibited KLK7 competitively in low micro-molar concentration (K_i_ = 3.6 μM). Using compound 8 to treat PANC-1 cells, the cell proliferation, migration and invasion abilities were significantly inhibited in a concentration-dependent manner. It is worth noting that similar results were obtained when compound 42 (another specific suicide inhibitor of KLK7 discovered previously) was used to treat PANC-1 cells. However, 8 is a reversible competitive inhibitor, and 42 is a irreversible suicide inhibitor that could have more side effects.

Altogether, our results suggest that KLK7 could be a chemotherapy target for the treatment of pancreatic cancer. The ten inhibitors of KLK7 with different molecular structures identified in this study could be used as lead compounds. The further optimization of these compounds could make them become effective clinical drugs for the treatment of pancreatic cancer.

## MATERIALS AND METHODS

### Clinic samples

The 49 pancreatic cancer tissues and corresponding adjacent normal pancreatic tissues, and 46 hepatocellular carcinoma tissues and corresponding adjacent liver tissues were obtained from Yichang Central People’s Hospital (The First College of Clinical Medical Science) of China Three Gorges University for research purposes (prior patients’ contents and the approval from the Ethics Committee of China Three Gorges University). The tissues were prepared and stored as formalin-fixed paraffin blocks.

### Immunohistochemistry

Formalin-fixed paraffin blocks of the clinic samples were sectioned (4 μm), deparaffinised (67°C, 2h) and rehydrated. After washed with 0.01 M phosphate-buffered saline (PBS, pH 7.4) for three times (3 min each time), the sections were placed in EDTA buffer (pH 9.0) and treated by high temperature (115°C) and pressure (171kPa) for 2 min and then washed two times (3min each time) in PBS. After treated with H_2_O_2_ (3%, v/v) for 10 min to quench endogenous peroxidase, the sections were incubated for 2 h with the rabbit anti-KLK7 polyclonal antibody (1:800 dilution, Novas) at room temperature. After washed three times (3 min each time) in PBS, the sections were then incubated for 40 min with the HRP mouse anti rabbit IgG antibody (EnVision) at room temperature and washed three times (3 min each time) again in PBS. Finally, the sections were incubated in 3,3′-diaminobenzidine (DAB) for 5 min, and counterstained with Mayer’s haematoxylin. Normal goat serum (10%) was used as the negative control in the place of the primary antibody.

### Microscopy imaging

The sections were observed under microscopy (Leica DMI3000B). Images were processed using Adobe Photoshop CS6 and integrated optical density (IOD) was measured using Image Pro Plus v6.0. The mean IOD was calculated from four images per section and used to represent the expression level of KLK7 protein in the tissues. In this experiment, two observers were blinded to the clinical data.

### shRNAs

The following four shRNAs were designed based on the human KLK7 gene sequence (Gen Bank, ID: 5650), and the specificity was verified using BLAST (PubMed): LV-hKLK7-shRNA-1,5′-GGCUGUCAUCCAUGGUGAAGAUUCAAGAGAUCUUCACCAUGGAUGACAGCCUUAGA-3′; LV-hKLK7-shRNA-2,5′-GACUGCACGAAGGUUUACAAGUUCAAGAGACUUGUAAACCUUCGUGCAGUCUU-3′; LV-hKLK7-shRNA-3,5′-GCAAGUUCACCAAGUGGAUAAUUCAAGAGAUUAUCCACUUGGUGAACUUGCUU-3′; LV-hKLK7-shRNA-4,5′-GCACUGAGUUAAUUAACUGUGUUUCAAGAGAACACAGUUAAUUAACUCAGUGCUU-3′. A scrambled shRNA sequence was used as the negative control (LV-NC-shRNA, 5′-UUCUCCGAACGUGUCACGUUUC-3′). The shRNA sequences were synthesized and cloned into recombinant shRNA expression vectors (pGLV3-H1-GFP-Puro). The resulted plasmids were verified by DNA sequencing.

### Preparation of lentivirus

To produce infective lentiviral particles, human embryonic kidney 293T cells were co-transfected with the lentivirus (LV) package helper plasmids (pGag/Pol, pRev and pVSV-G; GenePharma, Shanghai, China) and the recombinant LV-hKLK7-shRNA (or LV-NC-shRNA) plasmids using RNAi-Mate (GenePharma, Shanghai, China). After 72 h, cell culture media were collected, filtered (0.45μm) and concentrated by ultra-centrifugation. The titer of lentivirus was calculated based on the expression of green fluorescent protein (GFP) in 293T cell.

### Preparation of lentivirus infected cells

PANC-1 and HepG2 cell lines were obtained from the Chinese Type Culture Collection Center (Wuhan, China). PANC-1 cells were maintained in dulbecco’s modified Eagle medium supplemented with 10% fetal bovine serum, 100 units/ml penicillin G sodium, and 100 μg/ml streptomycin sulphate. HepG2 cells were maintained in RPMI 1640 medium supplemented with 10% fetal bovine serum, 100 units/ml penicillin G sodium, and 100 μg/ml streptomycin sulphate. PANC-1 and HepG2 cells pro-treated by 5 μg/ml polybrene were infected with the lentivirus and stably infected cells were selected in cell culture medium containing 4 μg/ml puromycin (Sigma-Aldrich).

### Real-time PCR

Total RNA from stably infected PANC-1cells was extracted using the RNA simple Total RNA Kit (DP419, TIANGEN Biotech, Beijing). The first-strand of the cDNA was synthesized from total RNA using Revert Aid™ First Strand cDNA Synthesis Kit (Thermo Fisher Scientific). Real-time PCR was performed with the following specific primers of KLK7: sense, 5′-ACCTCATGCTCGTGAAGCTC-3′; anti-sense, 5′-CCGGAGACAGTACAGGTGGT-3′. For GAPDH analysis (inner control), the following primers were used: sense, 5′-CAAGGTCATCCATGACAACTTTG-3′; anti-sense, 5′-GTCCACCACCCTGTTGCTGTAG-3′. Real-time PCR was performed on an Agilent Statagene Mx3000P machine using the SYBR Green II Dye detection system (SYBR^®^ Premix Ex Taq™ II, RR820A). Relative levels of gene expression were normalized against the level of GAPDH.

### Western blotting assay

Western blotting assay was performed with cell lysates collected from stably transfected cells. Total protein concentrations were measured with the bicinchoninic assay (BCA). The sample proteins (20μg for each lane) were analyzed first in 12% SDS-PAGE and then transferred onto polyvinylidine difuorides membranes. Membranes were blocked with 5% skim milk and then incubated with anti-KLK7 polyclonal antibody (1:3000 dilutions, Novas) at 4°C overnight. After incubated with horseradish peroxidase-conjugated goat anti-rabbit secondary antibody (1:3000, Goodbio Technology, Wuhan, China), the membranes were developed by ECL reagent (Goodbio Technology, Wuhan, China). The chemiluminescent data were collected and grayscale values were evaluated with Image J.

### Cell proliferation assay

Real time cell proliferation assays were performed on RTCA xCELLigence DP (ACEA Biosciences). Cells were seeded into 16-well E-Plate at a density of 5×10^3^ cells/well. Cell index data were recorded every 15 min for 72 h. At the end of the assay, the cells were photographed on a microscopy (Leica DMI3000B).

### Migration and Matrigel invasion assays

Cell migration assay was performed on RTCA xCELLigence DP (ACEA Biosciences). Cells were seeded into the upper chamber of CIM-Plate 16 at a density of 2 × 10^4^ cells/well. Migration was measured as the relative cell index change measured by the microelectronic sensors integrated into the bottom side of the membrane. Measurements were recorded every 15 min for 36 h.

Cell matrigel invasion assay was similar to the cell migration assay, except that the microporous membrane between the upper and lower chambers was coated by a thin layer of matrigel (BD Biosciences). After 36 h, cells on the bottom side were fixed in cold methanol, stained with 0.4% crystal violet, and photographed (5 random fields for each sample).

### The recovery assay by exogenous KLK7

For proliferation assays, the cell culture medium (conditional medium) were collected, concentrated and used as exogenous KLK7 to treat the indicated PANC-1 cells (5 × 10^3^ cells/well). The cell proliferation was evaluated using RTCA. For migration and matrigel invasion assays, indicated PANC-1 cells were seeded into the lower (4 × 10^3^ cells/well) and upper (2 × 10^4^ cells/well) chambers of CIM-Plate 16 respectively. The migration and invasion capacity of PANC-1 cells was evaluated using RTCA.

### Vitural screening of KLK7 inhibitors

The X-ray structure of KLK7 was retrieved from PDB (PDB ID: 2QXH) and analyzed in MOE to generate a pharmacophore model. The Chemdiv database was prepared in Pipeline Pilot to generate the 3-D conformations, and then filtered by Lipinski Rule of Five (Hydrogen bond donors = 5; Hydrogen bond acceptor = 10; Molecular weight = 120–600 Da; AlogP = 6; Number of violations Allowed = 1) and the pharmacophore modelin MOE (Enable Partial Match, at least 5). Structure-based screening experiments were then performed using a two-step protocol by the combination of GOLD and CLC Drug Discovery Workbench screening programs. The druggable regions of KLK7 were firstly identified by DoGSiteScorer, and the docking step was then carried out using GOLD (GA runs = 30; Fitness & Search option was CHEMPLP; GA Setting was Automatic-searching efficiency: 30%; Active site: CMK, 10Å) to generate a library containing molecules with appropriate shape complementarity with the search zone defined around Ser195 in the active site. The top 10% of the molecules ranked by GOLD (PLP.Fitness) were retained. Similar molecules were removed and the rest were then re-docked by CLC Drug Discovery Workbench docking software (Number of iterations for each ligand = 100).The top 300 hits ranked by CLC Drug Discovery Workbench (PLANTS_PLP_ score) were finally kept and purchased from Chemdiv for *in vitro* evaluation.

### Enzymatic assays

KLK7 activity were determined by monitoring the hydrolysis of the fluorogenic substrate (MCA-Arg-Pro-Lys-Pro-Val-Glu-Nval-Trp-Arg-Lys(Dnp)-NH_2_, λ_exc_ = 320, λ_em_ = 405 nm) for 1 h at 37°C. Substrates and inhibitors were dissolved in DMSO. The final concentration of KLK7 and the substrate were 7.6 nM and 10 μM respectively in the activity buffer (50 mM Tris-HCl, pH 8.0, Tween-20 0.01 % (v/v) and 150 mM NaCl). The reactions were incubated for 15 min before the test. Initial rates determined in control experiments (V_0_) were considered to be 100 % of the proteinase activity; initial rates (V_i_) that were below 100 % in the presence of a tested compound were considered to be inhibitions. The inhibitory activity of the compounds was expressed as IC_50_ (inhibitor concentrations giving 50% inhibition). The final concentration of DMSO in the test was 2% (v/v).

The reversibility of inhibition was evaluated by the dilution assay. The reaction mixtures were prepared by incubating KLK7 with the compounds for 15 or 60 min. Aliquots of the reaction mixtures (2.5 μL) were added to 97.5 μL of the activity buffer containing the fluorogenic substrate. The mechanism of inhibition was determined by varying substrate and inhibitor concentrations and using classical representations (Lineweaver-Burk).

### Inhibition of PANC-1 cells by KLK7 inhibitors

The effect of Compound 8 (a KLK7 inhibitor discovered by virtual screening described above) on PANC-1 was evaluated in this experiment. Compound 42 (a KLK7 covalent inhibitor discovered previously) was used as a positive control in this assay. Compound 42 is a coumarinic derivative, and can form irreversible covalent bonds with KLK7 to inhibit its activity [30]. The compound 42 was synthesized in the same way as reported [30], and its molecular structure and purity was confirmed by NMR and HPLC (Supplementary Figure 3). The tested compound was added into the cell culture medium 3 h after PANC-1 cells were seeded. The cell prolifiation, migration and matrigel invasion was then assayed on RTCA.

### Statistic analysis

All of the experiments above were assayed with three replicates and repeated three times. The data were represented as mean ± standard deviation, and the statistical analysis was performed using SPSS 18.0. The statistical analysis between two selected groups was performed with Student’s *t*-test, and the comparison of two groups in multiple groups was performed using one-way ANOVA with Tukey’s post hoc analysis. *P* values less than 0.05 were considered statistically significant.

## SUPPLEMENTARY MATERIALS FIGURES



## Abbreviations

KLKs: kallikrein-related peptidases
IHC: immunohistochemistry
shRNAs: short hairpin RNAs
KDs: PANC-1 cell lines with stable KLK7 knocking-down or PANC-1 cells infected by LV-hKLK7-shRNA
BC: untreated PANC-1 cells
NC: PANC-1 cells infected by control lentivirus LV-NC-shRNA
RTCA: real time cellular analysis
PDAC: Pancreatic ductal adenocarcinoma
ECM: extracellular matrix
CAMs: cellular adhesive molecules
DAB: 3,3′-diaminobenzidine
IOD: integrated optical density
LV: lentivirus

## References

[1] Torre LA, Bray F, Siegel RL, Ferlay J, Lortet-Tieulent J, Jemal A (2015). Global cancer statistics, 2012. CA Cancer J Clin.

[2] Krejs GJ (2010). Pancreatic cancer: epidemiology and risk factors. Dig Dis.

[3] Stathis A, Moore MJ (2010). Advanced pancreatic carcinoma: current treatment and future challenges. Nat Rev Clin Oncol.

[4] Yousef GM, Diamandis EP (2001). The new human tissue kallikrein gene family: structure, function, and association to disease. Endocr Rev.

[5] Yousef GM, Obiezu CV, Luo LY, Magklara A, Borgono CA, Kishi T, Memari N, Michael l P, Sidiropoulos M, Kurlender L, Economopolou K, Kapadia C, Komatsu N (2005). Human tissue kallikreins: from gene structure to function and clinical applications. Adv Clin Chem.

[6] Linardoutsos D, Gazouli M, Machairas A, Bramis I, Zografos GC (2014). Kallikrein-related peptidases in cancers of gastrointestinal tract: an inside view of their role and clinical significance. J BUON.

[7] Avgeris M, Mavridis K, Scorilas A (2010). Kallikrein-related peptidase genes as promising biomarkers for prognosis and monitoring of human malignancies. Biol Chem.

[8] Yousef GM, Scorilas A, Magklara A, Soosaipillai A, Diamandis EP (2000). The KLK7 (PRSS6) gene, encoding for the stratum corneum chymotryptic enzyme is a new member of the human kallikrein gene family - genomic characterization, mapping, tissue expression and hormonal regulation. Gene.

[9] Walker F, Nicole P, Jallane A, Soosaipillai A, Mosbach V, Oikonomopoulou K, Diamandis EP, Magdolen V, Darmoul D (2014). Kallikrein-related peptidase 7 (KLK7) is a proliferative factor that is aberrantly expressed in human colon cancer. Biol Chem.

[10] Dorn J, Gkazepis A, Kotzsch M, Kremer M, Propping C, Mayer K, Mengele K, Diamandis EP, Kiechle M, Magdolen V, Schmitt M (2014). Clinical value of protein expression of kallikrein-related peptidase 7 (KLK7) in ovarian cancer. Biol Chem.

[11] Iakovlev V, Siegel ER, Tsao MS, Haun RS (2012). Expression of kallikrein-related peptidase 7 predicts poor prognosis in patients with unresectable pancreatic ductal adenocarcinoma. Cancer Epidemiol Biomarkers Prev.

[12] Ramani VC, Haun RS (2008). The extracellular matrix protein fibronectin is a substrate for kallikrein 7. Biochem Biophys Res Commun.

[13] Johnson SK, Ramani VC, Hennings L, Haun RS (2007). Kallikrein 7 enhances pancreatic cancer cell invasion by shedding E-cadherin. Cancer.

[14] Ramani VC, Haun RS (2008). Expression of kallikrein 7 diminishes pancreatic cancer cell adhesion to vitronectin and enhances urokinase-type plasminogen activator receptor shedding. Pancreas.

[15] Ramani VC, Hennings L, Haun RS (2008). Desmoglein 2 is a substrate of kallikrein 7 in pancreatic cancer. BMC Cancer.

[16] Debela M, Hess P, Magdolen V, Schechter NM, Steiner T, Huber R, Bode W, Goettig P (2007). Chymotryptic specificity determinants in the 1.0 A structure of the zinc-inhibited human tissue kallikrein 7. Proc Natl Acad Sci U S A.

[17] Wong HH, Lemoine NR (2009). Pancreatic cancer: molecular pathogenesis and new therapeutic targets. Nat Rev Gastroenterol Hepatol.

[18] Silvestris N, Gnoni A, Brunetti AE, Vincenti L, Santini D, Tonini G, Merchionne F, Maiello E, Lorusso V, Nardulli P, Azzariti A, Reni M (2014). Target therapies in pancreatic carcinoma. Curr Med Chem.

[19] Patten LC, Berger DH (2005). Role of proteases in pancreatic carcinoma. World J Surg.

[20] Filippou PS, Karagiannis GS, Musrap N, Diamandis EP (2016). Kallikrein-related peptidases (KLKs) and the hallmarks of cancer. Crit Rev Clin Lab Sci.

[21] Borgono CA, Diamandis EP (2004). The emerging roles of human tissue kallikreins in cancer. Nat Rev Cancer.

[22] Michel N, Heuze-Vourc’h N, Lavergne E, Parent C, Jourdan ML, Vallet A, Iochmann S, Musso O, Reverdiau P, Courty Y (2014). Growth and survival of lung cancer cells: regulation by kallikrein-related peptidase 6 via activation of proteinase-activated receptor 2 and the epidermal growth factor receptor. Biol Chem.

[23] Lu Z, Yang Q, Cui M, Liu Y, Wang T, Zhao H, Dong Q (2014). Tissue kallikrein induces SH-SY5Y cell proliferation via epidermal growth factor receptor and extracellular signal-regulated kinase1/2 pathway. Biochem Biophys Res Commun.

[24] Jin Y, Qu S, Tesikova M, Wang L, Kristian A, Maelandsmo GM, Kong H, Zhang T, Jeronimo C, Teixeira MR, Yuca E, Tekedereli I, Gorgulu K (2013). Molecular circuit involving KLK4 integrates androgen and mTOR signaling in prostate cancer. Proc Natl Acad Sci U S A.

[25] Kostenuik PJ (2004). Revisiting the seed and soil theory of bone metastasis: new tools, same answer. J Musculoskelet Neuronal Interact.

[26] Kryza T, Silva ML, Loessner D, Heuze-Vourc’h N, Clements JA (2016). The kallikrein-related peptidase family: Dysregulation and functions during cancer progression. Biochimie.

[27] Tan X, Bertonati C, Qin L, Furio L, El Amri C, Hovnanian A, Reboud-Ravaux M, Villoutreix BO (2013). Identification by in silico and in vitro screenings of small organic molecules acting as reversible inhibitors of kallikreins. Eur J Med Chem.

[28] Zhang CY, Zhu Y, Rui WB, Dai J, Shen ZJ (2015). Expression of kallikrein-related peptidase 7 is decreased in prostate cancer. Asian J Androl.

[29] Leelananda SP, Lindert S (2016). Computational methods in drug discovery. Beilstein J Org Chem.

[30] Tan X, Soualmia F, Furio L, Renard JF, Kempen I, Qin L, Pagano M, Pirotte B, El Amri C, Hovnanian A, Reboud-Ravaux M (2015). Toward the first class of suicide inhibitors of kallikreins involved in skin diseases. J Med Chem.

