# Supplementary material for: Kallikrein-related peptidase 7 is a potential target for the treatment of pancreatic cancer
Source: Oncotarget. 2018 Jan 10;9(16):12894–906. doi: 10.18632/oncotarget.24132 (PMC5849182; doi:10.18632/oncotarget.24132)
Supplement: Supplementary file 1 [file oncotarget-09-12894-s001.pdf]

Kallikrein-related peptidase 7 is a potential target for the treatment of pancreatic cancer

SUPPLEMENTARY MATERIALS

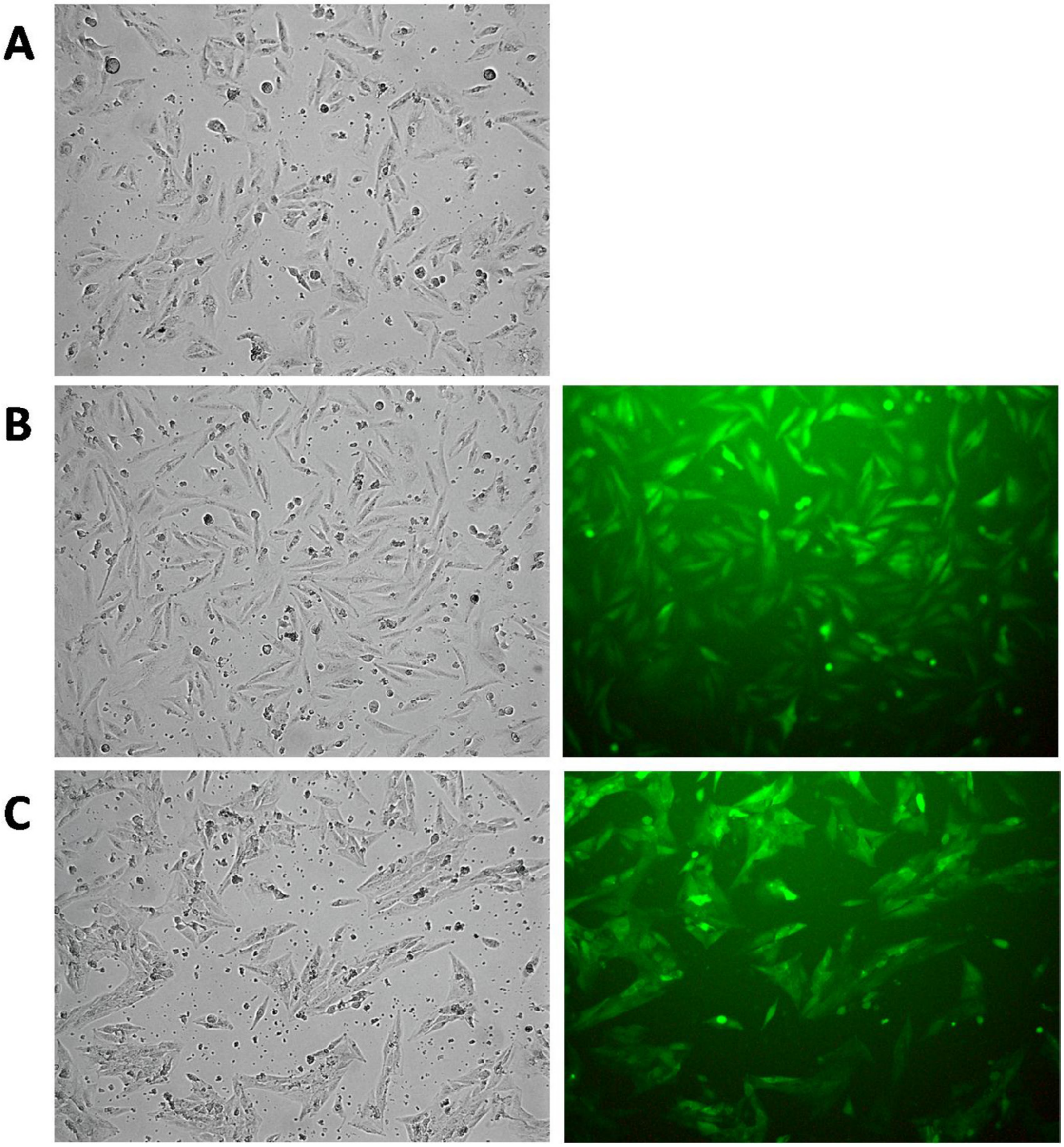

Supplementary Figure 1: Cell morphology of PANC-1 cells infected by lentivirus. (**A**) cell morphology of PANC-1 cells in BC group, which are uninfected PANC-1 cells; (**B**) cell morphology of PANC-1 cells in NC group, which are PANC-1 cells infected with lentivirus LV-NC-shRNA; (**C**) cell morphology of PANC-1 cells in KD4 group, which are PANC-1 cells infected with lentivirus LV-hKLK7-shRNA-4. KD4 cell morphology changes into fusiform shape, and easily gathered into group, indicating that KLK7 gene silencing increase the cellular adhesion and decrease cellular mobility.

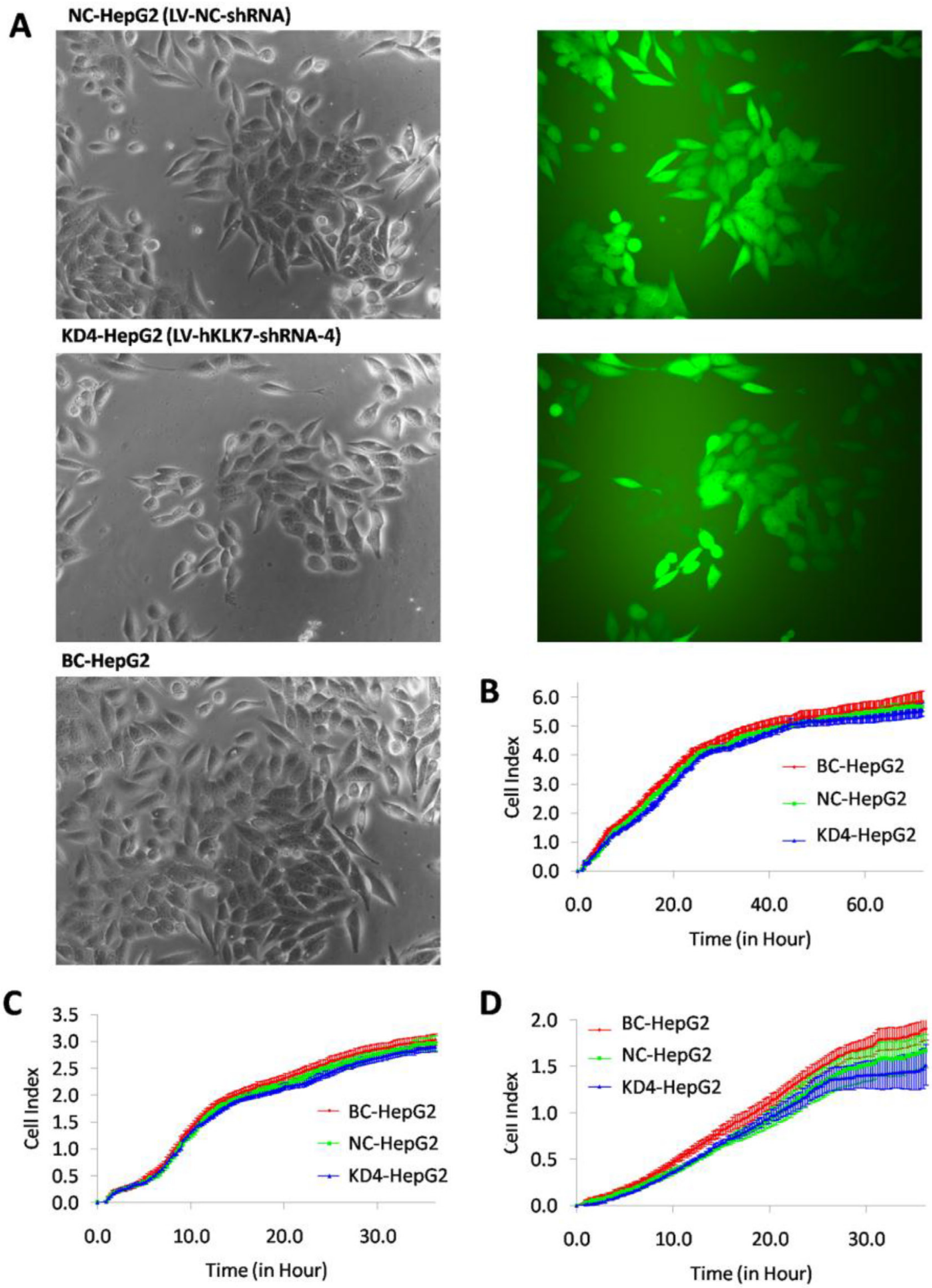

Supplementary Figure 2: Compact of KLK7 silencing on HepG2 cell proliferation, migration and invasion abilities identified by RTCA. (**A**) KD4-HepG2 and NC-HepG2 are HepG2 cells infected with lentivirus LV-hKLK7-shRNA-4 and LV-NC-shRNA, BC-HepG2 are uninfected HepG2 cells. (**B–C)** and (**D**) proliferation, migration and invasion curve of HepG2 cells in BC-HepG2, NC-HepG2 and KD4-HepG2 groups. Silencing KLK7 gene expression in HepG2 cell has not effect on HepG2 cell proliferation, migration and invasion abilities.

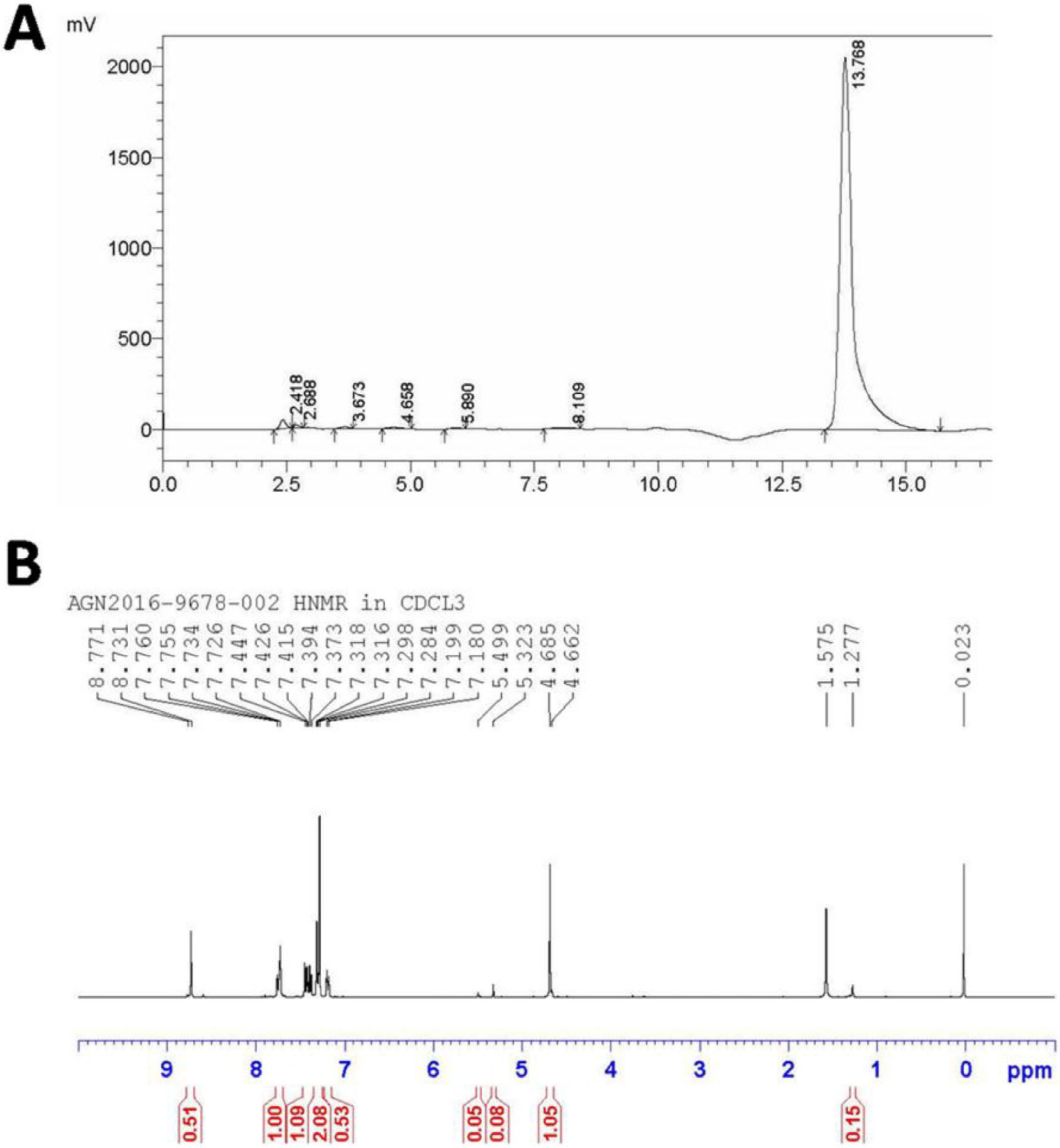

Supplementary Figure 3: Analysis of synthesized compound 42 using HPLC and NMR. (**A**) purity of compound 42 analyzed by HPLC, purity >95%; (**B**) Confirmation of molecular structure of compound 42 by NMR.
